# Association of Variable Number of Tandem Repeats in the Coding Region of the FAM46A Gene, FAM46A rs11040 SNP and BAG6 rs3117582 SNP with Susceptibility to Tuberculosis

**DOI:** 10.1371/journal.pone.0091385

**Published:** 2014-03-13

**Authors:** Godfrey Essien Etokebe, Ljiljana Bulat-Kardum, Ludvig Andre Munthe, Sanja Balen, Zlatko Dembic

**Affiliations:** 1 Molecular Genetics Laboratory Department of Oral Biology, Faculty of Dentistry, University of Oslo, Oslo, Norway; 2 Section of Pulmology, Department of Internal Medicine, Clinical Hospital Center, University of Rijeka, Rijeka, Croatia; 3 Dept of Immunology, OUS-Rikshospitalet, Oslo, Norway; 4 Clinical Institute for Transfusion Medicine, Universal Hospital Center Rijeka, School of Medicine, University of Rijeka, Rijeka, Croatia; St. Petersburg Pasteur Institute, Russian Federation

## Abstract

We analyzed for association between the Family with sequence similarity 46, member A (FAM46A) gene (located on chromosome 6q14.1), BCL2-Associated Athanogene 6 (BAG6) gene (located on chromosome 6p21.3) and tuberculosis in Croatian Caucasian. We genotyped the FAM46A rs11040 SNP, FAM46A VNTR and BAG6 rs3117582 polymorphisms in a case-control study with 257 tuberculosis patients and 493 healthy individuals in a Croatian Caucasian population. We found that genotype FAM46A 3/3 (three VNTR repeats homozygote) was associated with susceptibility to tuberculosis (p<0.0015, P_corr._<0.029, Odds ratio = 2.42, 95% Confidence Interval = 1.34–4.3). This association suggests that the protein domain encoded by the VNTR might be important for the function of the FAM46A protein, which, in turn, could be relevant in developing tuberculosis. In addition, we found that FAM46A rs11040 SNP:FAM46A VNTR:BAG6 haplotype 132 (G-3-C) is associated with susceptibility to tuberculosis (p<0.012, p_corr._<0.024, Odds ratio 3.45, 95% Confidence Interval = 1.26–9.74). This may suggests that the interaction between the FAM46A and BAG6 proteins may be involved in tuberculosis etiology. We found also that infection of human macrophages with heat-killed *M. tuberculosis* (H37Rv) led to over-expression of FAM46A (VNTR 3/4) transcript. This is the first study to show associations between the FAM46A gene VNTR polymorphisms, FAM46A rs11040 SNP:FAM46A VNTR:BAG6 haplotypes and any disease.

## Introduction

Tuberculosis (TB) is the leading killer among infectious diseases and constitutes a major health problem in developing world, with yearly incidences of around 8.7 million cases globally [1]. *Mycobacterium tuberculosis* (MTB) is the main cause of this disease. Although about 30% of the world population is exposed to MTB, only 10% of those exposed become infected. Remarkably, only half of the infected individuals develop clinical TB within two years of infection, while the other half of infected individuals may develop clinical TB later in life or maintain latent infection for the rest of their lives [2], [3]. MTB is a very resilient bacterium due to many factors including a significant ability to adapt to the environment and to inhibit phagosome maturation. Long latency, coupled with reactivation under conditions that weaken the immune system, also contributes to the resilience of MTB [4], [5].

In addition to the well established influence and involvement of social and environmental factors in conferring susceptibility to TB [6], genetic predisposition also plays a significant role [7]. Susceptibility to TB is complex (i.e. multigenic), as shown by hereditary and genetic association analyses, including those performed on adopted siblings [8], twins [9], genome-wide linkages [10], and population-based case-control studies [11]. The most widely used approach to investigate TB susceptibility is the candidate gene association study, and several genes have been found to be associated with MTB infection and/or disease. These include macrophage specific genes, such as the natural resistance-associated macrophage protein (NRAMP) gene [12], the interleukin 1 receptor antagonist (IL1RA) gene [13], and the lysosomal-associated membrane protein 1 (Lamp1) [14].

Most of the genetic association reports studying complex diseases use single nucleotide polymorphisms (SNPs) as markers. A genomic variant that is understudied is the variable number of tandem repeats (VNTR) [14], [15]. This may be due to VNTR complexity and the challenges in assaying them. VNTRs can modulate many biological processes, such as gene transcription, protein function, morphological development, behavior, and physiology. They may also be responsible for many disorders in humans that include unstable (genetic) repeat expansions [16], [17]. For the genetic epidemiology of tuberculosis, there are only two reports on the association between VNTRs (in the 5-lipoxygenase (ALOX5) and interleukin 1 receptor agonist (IL1RA) genes) and tuberculosis susceptibility in case-control studies [13].

The Family with sequence similarity 46, member A (FAM46A) gene was first identified and cloned from human retina tissue as a retinal disease candidate gene [18]. It is located at chromosome 6: 82,201,156–82,462,491. About five alternative splice variants of the FAM46A gene have been reported so far (http://www.ensembl.org/Homo_sapiens/Gene/Summary?g=ENSG00000112773;r=6:82201156-82462491). A striking characteristic of the FAM46A gene is the presence of a VNTR within its coding sequence in exon 2. This VNTR may vary from two to seven repeats per chromosome [19], [20] and can produce allelic variants that may be able to modulate many biological processes [16], [17]. This VNTR generates also protein modifications with a variable glycine-rich domain. The consensus coding sequence of the FAM46A gene is the splice variant, which is 5609 base pairs (bp) long and encodes a 442 amino-acid polypeptide and contains four VNTRs. The FAM46A polypeptide chain also contains the Domain of unknown function 1693 (DUF1693) [21]. Similarly, no biological role has been assigned to the FAM46A gene. Using yeast two hybrid systems, some proteins have been found to interact with the FAM46A protein [22], [23]. However, the biological significance of these interactions has not yet been elucidated. FAM46A protein interacts with the BAG6 protein [22], which is known to play a role in the early response to MT infection [24] by participating in apoptosis in macrophages. Also, it interacts with the zinc finger, FYVE domain-containing 9 (ZFYVE9) protein [22], which is involved in TGF-β signaling.

We previously reported that the mouse homologue of the gene (Fam46a) is expressed in developing tooth buds, and further suggested that, due to its nuclear localization and interaction with the human transcription factor, ZFYVE9 protein, Fam46a protein might be involved in cellular proliferation [25]. Therefore, we used a candidate gene approach to study the association of human FAM46A and BAG6 genes with tuberculosis. The FAM46A rs11040 SNP, FAM46A VNTR polymorphism and BAG6 rs3117582 SNP allowed us to analyze differences in their allelic, genotypic and haplotypic frequencies and search for a link with the disease using a case-control study of a Croatian Caucasian population.

## Results

### Allelic and Genotypic Frequencies of the FAM46A rs11040 SNP, FAM46A Gene VNTR and BAG6 rs3117582 SNP

**Figure 1:**
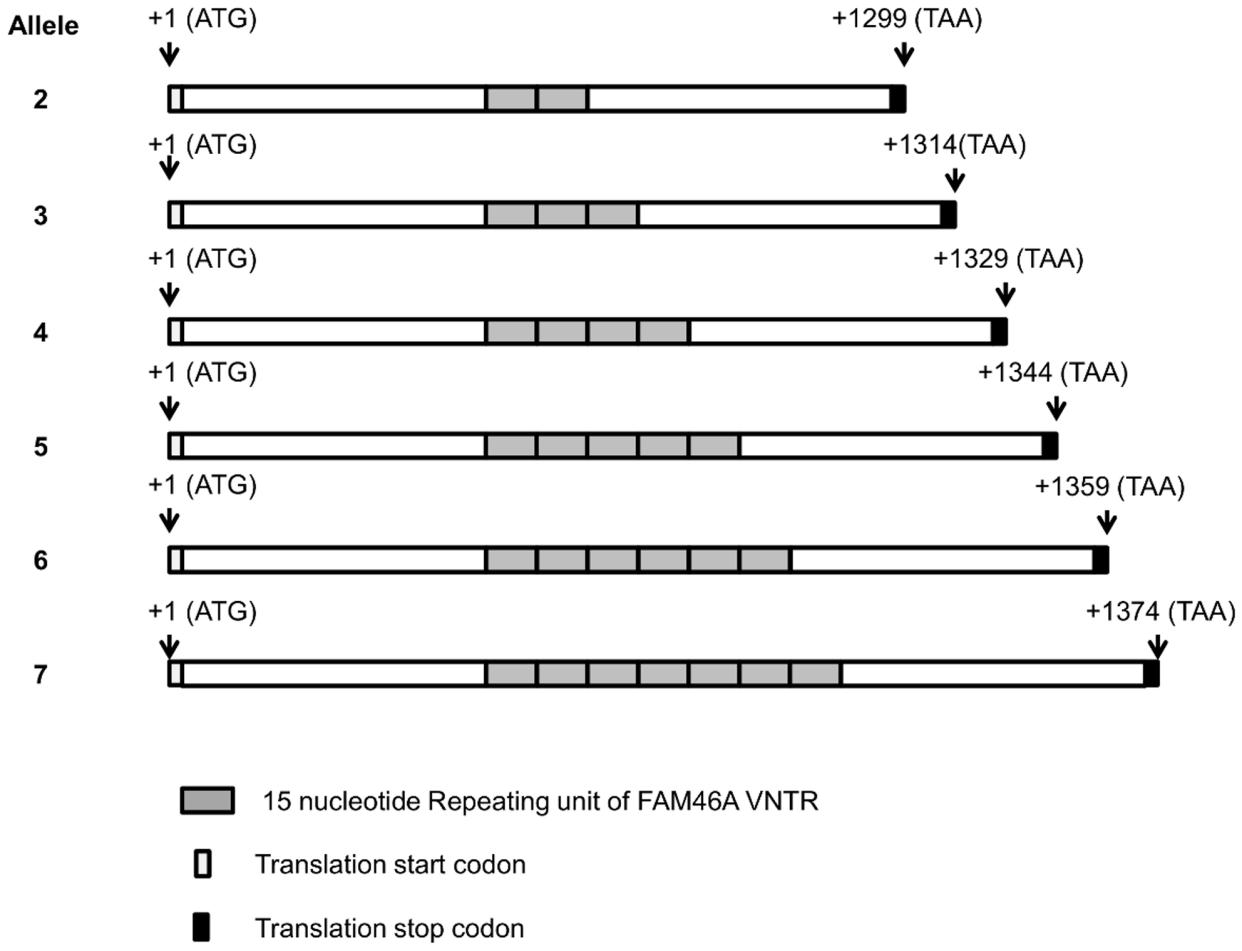
Schematic representation of FAM46A mRNA alleles in CCDS. Cloning and sequencing of FAM46A mRNA from our studied population generated six variants of the FAM46A gene that were different at the VNTR site. FAM46A alleles 3, 4, 5, and 6 (UniProt identifiers: >sp|Q96IP4|29-33), (>sp|Q96IP4|24-28), (>sp|Q96IP4|34-38) and (>sp|Q96IP4|39-43, respectively) were previously reported. Our submission of the sequences for FAM46A allele 2 and 7 to the GenBank has recently (06.02.2014) been accepted for publication with accession numbers KF878392 and KF878393, respectively.

The VNTR polymorphism in the FAM46A was genotyped in 257 tuberculosis patients and 450 control (healthy) individuals. All alleles and genotypes that we found were confirmed by re-typing and DNA sequencing in at least two independent samples for the respective genotype. We found six alleles (Figure 1) and nineteen genotypes for the FAM46A gene. We further compared VNTR allele frequencies in patients (n = 257) with normal healthy individuals (n = 450) and found that they were not different. We then compared genotypic VNTR frequencies between cases and controls. The genotype group 3/3 was significantly more frequent in cases than controls (p<0.0015, OR = 2.42, 95% CI = 1.4–4.3) and when the Bonferroni correction was applied (P_corr._ <0.029; Table 1). We genotyped also 249 patients and 486 normal healthy individuals for FAM46A rs11040 SNP polymorphisms and found that only the GG genotype (major allele homozygote) was present in both the patients and healthy control group (data not shown). We genotyped further our patient group (249) and normal healthy individuals group (486) for BAG6 rs3117582 SNP polymorphisms and compared genotypic and allelic frequencies in patients with the normal healthy individuals. We found that both the BAG6 rs3117582 SNP genotypes and alleles were not significantly different between the patients and the normal healthy control group (Table 2 and Table 3). When stratified by gender, the AA (major allele homozygote) genotype frequency was increased in the normal healthy male individuals group than in the male patients group (p<0.09, OR = 0.647, 95% CI = 0.0387–1.085). This difference did not reach statistical significance (data not shown).

**Table 1:** Association analysis of FAM46A gene VNTR genotypes with tuberculosis in Croatian Caucasian.

	Genotype frequency				
Genotype (VNTR)^a^	Patients (n = 257) frequency (N)	Controls (n = 450) frequency (N)	p-value	OR (95% CI)	Association
3/3	0.13 (32)	0.06 (25)	0.0015^b^	2.42 (1.34–4.3)	Susceptibility
4/4	0.05 (12)	0.05 (21)	1.0		–
5/5	0.17 (43)	0.16 (73)	0.92		–
6/6	0 (1)	0 (2)	1.0		–
7/7	0.01 (2)	0 (1)	0.56		–
2/3	0	0.01 (4)	0.3		–
2/4	0	0 (1)	1.0		–
2/5	0 (1)	0.01 (4)	0.66		–
2/6	0	0 (1)	1.0		–
3/4	0.10 (25)	0.12 (53)	0.46		–
3/5	0.22 (57)	0.24 (107)	0.64		–
3/6	0.02 (6)	0.03 (15)	0.5		–
3/7	0 (1)	0.01 (5)	1.0		–
4/5	0.18 (47)	0.20 (97)	0.49		–
4/6	0.04 (9)	0.02 (11)	0.48		–
4/7	(0) 1	0	1.0		–
5/6	0.06 (16)	0.07 (30)	0.88		–
5/7	0.01 (3)	0.01 (4)	0.71		–
6/7	0 (1)	0 (1)	1.0		–

N: number of alleles per group, n: total number of samples, OR: Odds ratio, CI: confidence interval, ^a^Integers represent the number of VNTR repeats,^ b^p_corr._<0.029.

**Table 2:** Association analysis of BAT3 gene rs311782 SNP genotypes with tuberculosis in Croatian Caucasian.

	Genotype frequency				
Genotype (VNTR)	Patients (n = 249) frequency (N)	Controls (n = 486) frequency (N)	p-value	OR (95%CI)	Association
A/A	0.84 (210)	0.87 (425)	0.26		–
C/C	0.01 (3)	0.01 (3)	0.41		–
A/C	0.14 (36)	0.12 (58)	0.35		–

N: number of genotypes per group, n: total number of samples, OR: Odds ratio, CI: confidence interval.

**Table 3:** Association analysis of BAT3 gene rs311782 SNP alleles with tuberculosis in Croatian Caucasian.

	Allele frequency				
Genotype (VNTR)	Patients (n = 498) frequency (N)	Controls (n = 972) frequency (N)	p-value	OR (95%CI)	Association
A	0.92 (456)	0.93 (908)	0.20		–
C	0.08 (42)	0.07 (64)	0.20		–

N: number of alleles per group, n: total number of samples, OR: Odds ratio, CI: confidence interval.

### Haplotype Analysis

We predicted the haplotypes by combining FAM46A rs11040 SNP, FAM46A VNTR and BAG6 rs3117582 SNP polymorphisms from 251 tuberculosis patients and 493 healthy control individuals. These three markers are separated by 76 basepairs and 50.8 megabase pairs (Mbp) on chromosome 6, respectively. We compared frequencies of haplotypes estimated from the three markers between tuberculosis patients and normal healthy individuals group and found that FAM46A rs11040 SNP:FAM46A VNTR:BAG6 haplotype 132 (G-3-C) was more frequent in the tuberculosis patients group than in the normal healthy control group (p = 0.012, p_corr._ = 0.024, Odds ratio: 3.45, 95% Confidence Interval = 1.26–9.74) (Table 4). Furthermore, genotype analyses of predicted haplotypes showed two significantly different genotypes (data not shown). These include a homozygote 132/132 (G-3-C/G-3-C) FAM46A rs11040:FAM46A VNTR:BAG6 rs3117582 genotype that was associated with susceptibility to tuberculosis (p_corr_ = 0.01; Odds Ratio: 2.26; 95% Confidence Interval: 1.21–4.23), whereas a heterozygotic genotype comprising of 132 and 151 haplotypes (G-3-C/G-5-A) was significantly associated with protection to disease (p_corr_ = 0.022; Odds Ratio: 0.62; 95% Confidence Interval: 0.42–0.91).

**Table 4:** Association analysis of FAM46A rs11040SNP:FAM46A VNTR:BAG6 gene rs3117582 SNP haplotypes with tuberculosis in Croatian Caucasian.

		Haplotype frequency					
Haplotype (Fam46A rs11040^a^:Fam46A VNTR^b^:BAG6 rs3117582^c^)	Other^d^	Patients (n = 502 frequency (N)	Controls (n = 986) frequency (N)	p-value	OR (95% CI)	Association	
121	G-2-A	(0.01) 2	(0.01) 9	0.35		–	
131	G-3-A	(0.56) 140	(0.27) 270	0.9		–	
132	G-3-C	(0.05) 12	(0.01) 7	0.012*	3.45 (1.26–9.74)	Susceptibility	
141	G-4-A	(0.39) 98	(0.02) 196	0.89		–	
142	G-4-C	(0.01) 3	(0.01) 5	1		–	
151	G-5-A	(0.73) 182	(0.39) 387	0.26		–	
152	G-5-C	(0.09) 23	(0.04) 40	0.68		–	
161	G-6-A	(0.12) 31	(0.05) 48	0.33		–	
162	G-6-C	(0.01) 2	(0.01) 14	0.1		–	
171	G-7-A	(0.02) 6	(0.01) 6	0.23		–	
172	G-7-C	(0.01) 3	(0.00)4	0.7		–	

N: number of haplotypes per group, n: total number of haplotypes, OR: Odds ratio, CI: confidence interval, ^a^1 represent major allele of FAM46A rs11040, ^b^Integers represent the number of ‘VNTR’ repeats,^c^1 represent major allele of BAG6 rs3117582 SNP while ^c^2 represent minor allele of BAG6 rs3117582 SNP, ^d^ Nucleotide designation of FAM46A rs11040 SNP-FAM46A VNTR copy number-Nucleotide designation of BAG6 rs3117582 SNP, * = P_corr._ = 0.024.

### Family with Sequence Similarity 46, Member A (Fam46A) Gene Expression in Macrophages after Infection with Heat-killed *M. Tuberculosis*

**Figure 2:**
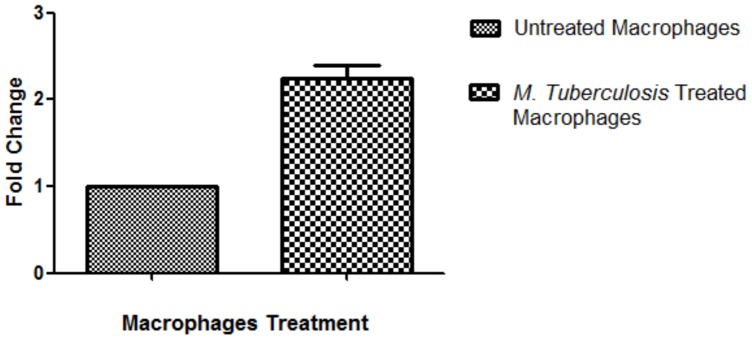
*Mycobacterium tuberculosis* (H37Rv) infection induces the over-expression of FAM46A RNA in macrophages. Total RNA was extracted from macrophages that were infected with *M. tuberculosis* (H37Rv) for 12 hours and uninfected macrophages, respectively. Extracted RNAs were subjected to quantitative real-time PCR for FAM46A gene amplification. Copy number was normalized to that of uninfected macrophages.

To investigate for a possible role for FAM46A protein in the modulation of macrophages response after infection with *M. tuberculosis*, we examined the effect of infecting human macrophages with heat-killed *M. tuberculosis* (H37Rv) and FAM46A RNA expression in macrophages. Because of the association that we found between FAM46A VNTR and susceptibility to tuberculosis, we postulated that it may have an effect on macrophage function. Here, we report the up-regulation of FAM46A RNA transcripts in macrophages upon infection with heat-killed *M. tuberculosis* (H37Rv) (Figure 2). Family with sequence similarity 46, member A (Fam46A) gene transcripts was increased in macrophages, with a mean fold change (SD) of 2.25 (±0.21), after 12 hours of infection with heat-killed *M. tuberculosis*. The genotype of VNTR alleles of the FAM46A gene expressed in macrophages was 3/4.

## Discussion

The present study characterizes epidemiologically the association between genetic variants in exon 2 of the FAM46A gene, a genetic marker in BAG6 gene and tuberculosis in a case-control study in a Croatian Caucasian population. The use of SNPs as markers for genetic association studies has facilitated the establishment of a technological platform known as genome wide association studies (GWAS). This platform allows for evaluation of thousands of SNP genetic markers together. Due to the complexity, challenges and lack of a technological platform that allows for the simultaneous assessment of VNTRs and SNPs, we assayed these genetic markers individually in our study.

We found that the homozygous carriers of three VNTR repeats of FAM46A gene (designated 3/3) were strongly associated with susceptibility to tuberculosis (Table 1). There is the possibility that this polymorphism could be a marker for a susceptibility factor not encoded by the FAM46A gene (i.e. miRNA, CpG island, or other gene in its vicinity). Additionally, these results need to be confirmed in other populations/groups. To date, this is the first report of an association between the FAM46A gene VNTR polymorphisms and any disease [19], [20]. We analyzed also for association between the FAM46A rs11040 SNP and tuberculosis in our case-control study and found that only the major allele was present in our study population. The rs11040 SNP is located 76 bp away from the 5′-end of the FAM46A VNTR start site. Our result (in conjunction with the close proximity of rs11040 SNP to FAM46A VNTR region) suggests that this region of the FAM46A gene may be highly conserved in Croatian Caucasian.

The function of the FAM46A gene and its component domains (PS50315 and DUF1693) is unknown [19]. The DUF domain is present in many hypothetical proteins including nematode prion-like proteins [21] and in nucleotidyltransferase superfamily genes with unknown function [21]. Our results suggest that the VNTR-encoded PS50315 domain of the FAM46A protein might have functional importance during MTB infection.

The FAM46A gene might have a role in TGF-β signaling and/or cell death (apoptosis). An experimentally determined interacting partner of the FAM46A protein is the ZFYVE9 protein [19], [22], [26], which is involved in the recruitment of unphosphorylated forms of SMAD2/SMAD3 to the TGF-β receptor (R) [27]. Phosphorylation of SMAD2/SMAD3 induces dissociation from ZFYVE9, and consequent formation of SMAD2/SMAD4 complexes, which translocate to the nucleus. Perhaps the FAM46A protein is involved in this cascade of events.

Another interacting partner of FAM46A is the BAG6 protein. This protein is known to play a role in ricin-induced apoptosis [28]. Recently, it has been shown that BAG6 plays a role in the early immune response to MT infection by regulating apoptosis induced by the *Mycobacterium tuberculosis*-derived protein ESAT-6 (Rv3875). This cascade of events also involves the anti-apoptotic BCL-2 protein [24]. BAG6 is a key protein associated with activation [29] and cell death [24] of antigen presenting cells during infection. It is tempting to speculate that BAG6 might be regulated through its interaction with FAM46A, perhaps in a manner that depends on the VNTR-encoded PS50315 domain. We found that FAM46A rs11040 SNP:FAM46A VNTR:BAG6 rs3117582 SNP haplotype 132 (G-3-C) conferred susceptibility to tuberculosis in the present study. This finding supports the notion that physical interactions between the protein products of genes on the same chromosome may play a vital role in their diseases’ association efficacy. In the present study, we have also shown that infection of macrophages with heat-killed *M. tuberculosis* (H37Rv) led to up-regulation of FAM46A RNA while a previous report also showed up-regulation of BAG6 RNA in macrophages upon stimulation with ESAT protein of *M. tuberculosis* [24]. Thus, we speculate that the FAM46A and BAG6 proteins interaction [22] may play an important role in tuberculosis etiology.

VNTRs in other genes are mostly outside protein-coding regions (open-reading frames), but they might be involved in different types of gene-controlling events. For example, it was previously reported that VNTRs in the IL1RA and ALOX5 genes might be associated with susceptibility to tuberculosis in humans [13], [30]. In both genes, the VNTRs are located in the promoter regions, and probably regulate the expression of mRNAs. As a result, these VNTRs are likely involved primarily in regulating the expression levels of proteins through their interaction with transcription factor(s) during gene transcription. In general, the location of VNTRs in the human genome is not constrained to the promoter regions of genes. They may be also found in exons, introns, and intergenic regions. This varied distribution of VNTR has, therefore, been postulated to provide a description for their involvement in the modulation of a variety of molecular processes [17]. In retrospect, this regulation may lead to functionally significant variability at cellular and system levels.

In conclusion, we have shown that the three-repeat homozygous variant (3/3) of the FAM46A gene VNTR, which is found in about 13% of those with the diseased, is associated with susceptibility to tuberculosis in the Croatian Caucasian population. We have also shown that a FAM46A rs11040 SNP:FAM46A VNTR:BAG6 haplotype is associated with susceptibility to tuberculosis. In addition, we have shown that the FAM46A RNA is over-expressed in macrophages upon infection with heat-killed *M. tuberculosis*. The FAM46A gene could therefore be a potential candidate that determines the risk of developing pulmonary tuberculosis in some individuals on its own or in association with other genes such as BAG6. This is the first report to show an association between a FAM46A VNTR, FAM46A rs11040 SNP:FAM46A VNTR:BAG6 haplotype and any disease.

## Materials and Methods

### Patients and Controls

The ethics committee of the Medical Research Council at the Medical faculty at the University of Rijeka approved this study. Patients were diagnosed, treated, and recovered at the Clinic for Internal Medicine (Clinical Hospital Center, Rijeka, Croatia) as previously reported [31]. Clinical diagnosis was supported by identification of *M. tuberculosis* from sputum by an *in vitro* culturing procedure. The total number of patients analyzed was 257, with 80% being males. This reflects the sex distribution of patients admitted at the tuberculosis treatment center in Rijeka. The mean (±SD) age of the patients was 51.03 (±15.71) years. For the VNTR analyses, the number of controls was 450, with 74.6% being males. The mean age of the patients was 41.84 (±11.90) years. Including other typings (FAM46A rs11040 and BAG6 SNPs), the total number of controls increased to 493. However, not all controls were typed for all three markers due to lack of particular samples. This was compensated by haplotype prediction assembly using Phase 2.1 software [32], [33]. However, the predictions for VNTR alleles of FAM46A, as deduced from haplotype analysis, were only used for haplotype-predicted analyses (Table 4), and not for statistical analyses in Tables 1 and 2 and 3. Healthy control subjects were unrelated blood donors that gave blood to the Department of Transfusion Medicine at the Clinical Hospital Center Rijeka. Tuberculosis skin test (PPD skin test) or quantiferon status was not assessed in the healthy control subjects. Both tuberculosis patients and normal controls were Croatian Caucasians matched for age and socioeconomic status (sex match was off by 4.57%). We, therefore, believe that confounding factors, including putative and causal variables, were most likely distributed equally among the two groups in our studied population. Both patients and controls provided oral and written consent.

### Genomic DNA Isolation, *in* *vitro* DNA Amplification, and VNTR Genotyping by DNA-sequencing Capillary Electrophoresis

DNA was isolated from frozen blood by a standard method, as previously described [31]. DNA fragments of 647 base pairs (bp) in length encoding the FAM46A gene were amplified from human genomic DNA by using FAM-labeled forward primers, designated Gfam_VF (5′-AGGGTACTTCGCCATGTCTG-3′), in combination with an unlabeled reverse primer, designated GEX_R (5′-CTCGTGATGGCCACAGATT-3′), by polymerase chain reaction (PCR). The 25 µL total volume PCR mixtures contained the following: 25 ng of genomic DNA, 0.2 µm each of the specific primers, and 1x Paq5000 Hotstart PCR master mix (Agilent Technologies, Inc., CA, USA). PCR was performed in a Peltier Thermal cycler (MJ Research, Massachusetts, USA). The Paq5000 polymerase was activated by an initial step at 95°C lasting 2 min, followed by 35 cycles of denaturing, annealing, and extension steps at 95°C for 20 s, 65°C for 20 s, and 72°C for 30 s, respectively, followed by a final extension step at 72°C for 5 min. Amplicons were resolved by 1% ethidium bromide-stained agarose gel electrophoresis and visualized by the Geldoc imaging system (Bio-Rad, Hercules, CA, USA). Amplicons (0.5 µl) were mixed with 0.5 µl GeneScan™ 1200 LIZ® Size Standard (Life Technologies, NY, USA) and loaded onto a 3730 DNA Analyzer (Life Technologies, NY, USA) for allele separation. Separated alleles were analyzed by the Genemapper software (Life Technologies, NY, USA). Allele (VNTR) identity was confirmed by sequencing directly PCR amplicons from samples that were genotyped as various homozygotes (two each). Also, randomly selected samples that were genotyped as heterozygotes were subcloned into TOPO Zero Blunt Sequencing plasmids (Life Technologies, NY, USA) prior to sequencing. Sequencing reaction was performed using the BigDye chemistry 3.1 (Life Technologies, NY, USA) with forward and reverse primers GVF (5′-AGGGTACTTCGCCATGTCTG-3′) and GEX_R (5′-CTCGTGATGGCCACAGATT-3′), respectively and resolved by the ABI 3730 DNA analyzer (Life Technologies, NY, USA).

### Genotyping of Single Nucleotide Polymorphisms

Family with sequence similarity 46, member A (Fam46A) gene rs11040 SNP and BAG6 gene rs3117582 SNP were assessed by probe-based real-time PCR assays as described by the Kits manufacturer (Life Technologies, NY, USA) in our tuberculosis patients and normal healthy individuals group, respectively. Stratagene MX3005 real-time PCR cycler was applied (Agilent Technologies, Santa Clara, CA, United States) for temperature cycling and signal quantification.

### Family with Sequence Similarity 46, Member A (Fam46A) Gene Expression in Macrophages after Infection with Heat-killed *M. tuberculosis* Infection

Venous blood was obtained from a normal healthy individual genotyped as FAM46A rs11040 SNP:FAM46A VNTR:BAG6 genotypes: G/G:3/4:A/A. Plasma blood mononuclear cells (PBMC) were extracted from the venous blood by ficoll gradient centrifugation using the Lymphoprep™ solution (Frensenius Kabi Norge AS, Oslo, Norway). Monocytes were isolated from 4×10^7^ PBMC using Dynabeads® CD14 magnetic beads (Life Technologies, NY, USA). Monocytes-dynabeads complexes (1×10^6^ cells/ml per aliquot) were cultured further in RPMI medium supplemented with 10% fetal bovine serum for four days prior to infection with heat-killed *M. tuberculosis* (H37Rv). Macrophages-dynabeads complexes were thereafter infected with 10 µl of high density heat-killed *M. tuberculosis* (H37Rv) suspension (1:1) per 1×10^6^ cells/ml aliquot for twelve hours. Total ribonucleic acid (RNA) was extracted from the heat-killed *M. tuberculosis* (H37Rv) infected macrophages and uninfected macrophages by the PureLink™ RNA Mini kit protocol (Invitrogen.com, Carlsbad, Ca, USA), respectively. Family with sequence similarity 46, member (FAM46A) RNA level in both the *M. tuberculosis* (H37Rv) infected macrophages and uninfected macrophages were assessed by quantitative real-time PCR using Hs_FAM46A_FAM_1 Quantifast Probe Assay kit (Qiagen, (Hilden, Germany) in 20 ng of total RNA per replicate. All assays were performed in triplicates. The result was reproduced in two independent experiments.

### Statistical Analysis

Power calculation was performed with the OPENEPI software (available at http://www.sph.emory.edu/~cdckms/sample%20size%202%20grps%20case%20control.html). Allelic and genotypic differences were analyzed by the chi-square (Fisher two tailed) method using the 2-way Contingency Table Analysis (available at http://statpages.org/ctab2x2.html and http://research.microsoft.com/en-us/um/redmond/projects/mscompbio/fisherexacttest/) between patients and controls. The Hardy-Weinberg analysis was done using the Arlequin software version 3.5 (Genetics and Biometry Laboratory, University of Geneva, Geneva, Switzerland), which showed that the FAM46A VNTRs and the BAG6 genotypes were in Hardy-Weinberg equilibrium (HWE). A statistically significant difference was defined when p was <0.05. Bonferroni correction (p_corr._) was performed by multiplying the p-value by 19 for the FAM46A VNTRs. The prediction of haplotypes for the FAM46A rs11040 SNP, FAM46A VNTRs and BAG6 rs3117582 SNP was done by the PHASE 2.1 software [32], [33]. For haplotypes, Bonferroni correction (p_corr._) was applied by multiplying p values by 2, since there were two loci assayed (FAM46A and BAG6). Namely, FAM46A rs11040 SNP was in total linkage disequilibrium with the FAM46A VNTR. FAM46A VNTRs and BAG6 haplotypes were in HWE.

In assessing the risk for tuberculosis, we wanted to estimate the strength and limitations of our study. The power of detection of significant differences of more than 5% in frequencies of VNTR genotypes was >84.5% (calculated by Kelsey, Fleiss, and Fleiss with continuity correction (CC) factor models). This analysis gave an OR 2.41 (alpha (α) = 0.05) with our sample size (case-control ratio = 0.58).
